# Comparative Characterisation of Subcutaneous, Intraperitoneal and Intrabursal OVCAR3 Models

**DOI:** 10.3390/ijms27187981

**Published:** 2026-09-08

**Authors:** Laura R. Moffitt, Jennie Do, Brittany R. Doran, Maree Bilandzic

**Affiliations:** 1 Hudson Institute of Medical Research, Melbourne, VIC 3168, Australia; jennie.do@monash.edu (J.D.); brittany.doran@monash.edu (B.R.D.); maree.bilandzic@hudson.org.au (M.B.); 2 Department of Molecular and Translational Science, Monash University, Melbourne, VIC 3168, Australia

**Keywords:** high-grade serous ovarian carcinoma, OVCAR3, xenograft, tumour microenvironment, orthotopic model, metastasis

## Abstract

OVCAR3 cells are widely used to establish animal models of high-grade serous ovarian cancer (HGSOC) via subcutaneous (SC), intraperitoneal (IP), and intrabursal (IB) routes. However, direct comparisons of tumour development across these implantation routes are limited. This study compared the establishment, progression, dissemination and histological characteristics of SC, IP and IB OVCAR3 xenografts in female athymic nude mice over defined route-specific assessment periods. Tumour development was evaluated using semi-quantitative tumour scoring, histological assessment, nuclear morphometry and immunofluorescence staining for PAX8, WT1 and Ki67. IP and IB routes achieved 100% engraftment whereas SC implantation achieved engraftment in 15 of 18 animals. IP produced rapid carcinomatosis by Week 2, IB produced localised ovarian growth before contralateral spread, and SC produced confined stromal-encapsulated tumours. Mechanical confinement in the IB niche increased intratumoural density (1087 cells/mm^2^) and compressed single-cell nuclear area (22 µm^2^) compared to IP/SC models. Histological and immunofluorescence assessment demonstrated tumour expression of PAX8, WT1 and Ki67 across the three models, with route-associated differences in tumour organisation and staining distribution. Nuclear morphometric measurements varied among the models, although the between-route differences were not statistically significant. Together, these findings define the principal characteristics and progression patterns of three commonly used OVCAR3 implantation models and provide a practical framework for selecting the route and experimental timeframe most appropriate to the aims of future studies.

## 1. Introduction

HGSOC remains the most lethal gynaecological malignancy, defined by rapid peritoneal dissemination, chemoresistance and late-stage diagnosis [1]. The near-universal recurrence following platinum-based chemotherapy creates a persistent demand for preclinical models that represent defined aspects of disease progression and the tumour microenvironment (TME) [2]. Patient-derived xenografts (PDX) offer biological complexity but have inherent variability and limited throughput [3]. Cell line-derived xenografts (CDX) remain widely used because of their experimental tractability, but their behaviour may differ according to the implantation protocol. Anatomical context changes the stromal, immune and physical environment encountered by implanted cells and may therefore be associated with differences in tumour establishment and progression [4,5].

Despite their common use, these models are reported with significant inconsistency regarding engraftment success and disease progression. While some studies report 100% take rates for OVCAR3 via the intraperitoneal route, others characterise its tumorigenicity as intermediate or variable, particularly in subcutaneous and intrabursal environments [4,6]. Many studies omit engraftment failures entirely, obscuring the inherent variability of specific implantation routes, hampering reproducibility of preclinical data, and concealing the real cost and animal burden associated with less efficient models. Furthermore, most studies lack the time-resolved analysis necessary to define progression rates or optimal windows for therapeutic intervention. This variation can make it difficult to compare tumour development across studies and to determine suitable cohort sizes and experimental endpoints. A time-resolved comparison of commonly used implantation routes would therefore assist both model selection and study planning.

These reporting gaps are important because the three routes are not interchangeable; each captures a distinct stage of HGSOC biology [4,5]. SC implantation is technically straightforward and suited to pharmacological screening but does not recapitulate peritoneal dissemination. IP injection mimics advanced carcinomatosis and malignant ascites but bypasses the early stages of tumour establishment and spread entirely, depositing cells directly into an environment that already represents end-stage disease. IB implantation places cells within the native ovarian niche but is technically demanding, with lower and more variable engraftment rates than either the IP or SC route. When successful, it produces locally confined disease with a defined window before peritoneal spread occurs. While OVCAR3 harbours key clinical HGSOC features such as *TP53* mutations, the degree to which local anatomical niches influence HGSOC lineage identity, spatial marker topology and cellular biophysics in vivo remains poorly defined. Histology, PAX8 and WT1 staining can be used together to assess tumour phenotype across these settings, and Ki67 provides an indication of proliferative activity. Comparison of these markers across implantation routes can show whether their expression and distribution are retained in the resulting tumours.

To address this, we compared OVCAR3 xenografts established by SC, IP and IB routes in female athymic nude mice. Route-specific longitudinal observations and tumour scoring were combined with anatomical mapping, histology, nuclear morphometry and immunofluorescence for PAX8, WT1 and Ki67. The resulting comparison describes tumour establishment and progression across the three protocols and provides practical information for model selection and experimental planning.

## 2. Results

### 2.1. Engraftment, Lineage Fidelity and Site-Specific Tumour Architecture Across Three Implantation Routes

**Figure 1:**
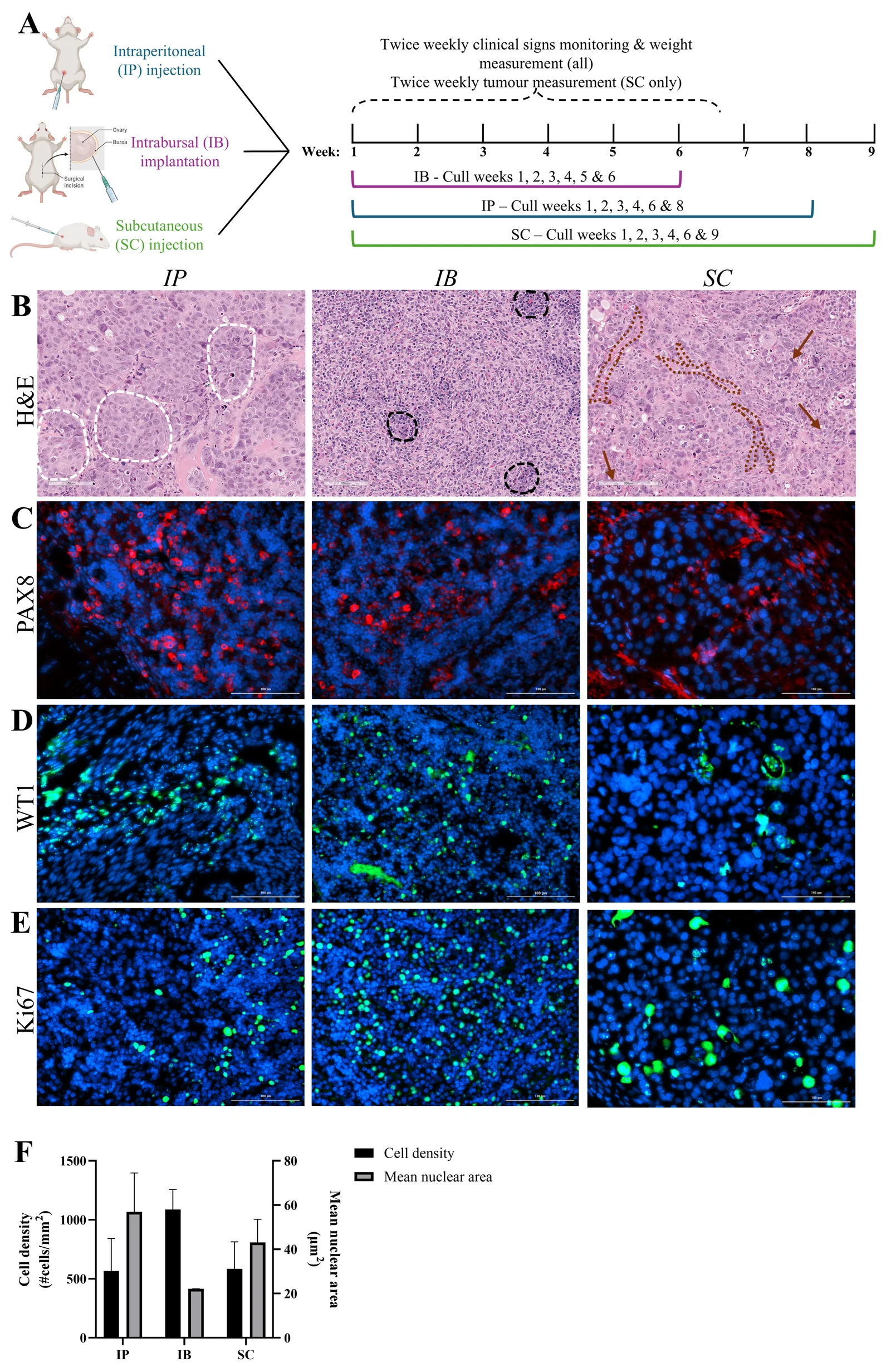
Experimental workflow, HGSOC lineage preservation and single-cell morphometry across anatomical niches. (**A**) Study design and experimental workflow. OVCAR3 cells were implanted into recipient mice via three distinct anatomical routes: intraperitoneal (IP), intrabursal (IB), and subcutaneous (SC). Monitoring parameters included longitudinal body weight and clinical tumour scoring, with staggered study endpoints (Weeks 1–9) optimised for the characteristic progression of each site. At termination, tissues were harvested for comprehensive pathological and molecular analysis. (**B**–**E**) Comparative histological and immunofluorescence characterisation. Representative images of tumour morphology and biomarker expression across the three implantation sites. (**B**) H&E staining illustrates site-specific growth patterns and morphology; the IP model displays aggressive, disseminated nodules displaying characteristic dense globular morphology (white dashed outlines); the IB model exhibits localised, cohesive growth retaining distinct glandular structures within the ovarian stroma (black dashed outlines); the SC model shows a confined mass featuring prominent internal fibrotic bands/strings (brown dotted outlines) and sporadic infiltrates of large stromal cells (brown arrows). All models maintain high-grade features, including nuclear atypia and high mitotic activity. (**C**) PAX8 expression (red) confirms Müllerian origin, with ubiquitous and strong nuclear positivity observed across all models. (**D**) WT1 expression (green) confirms the maintenance of the high-grade serous phenotype regardless of the anatomical niche. (**E**) Ki67 expression (green), demonstrating a high proliferative capacity across all implantation sites. Nuclei were counterstained with DAPI (blue). Scale bars = 100 µm (20× magnification). (**F**) Quantitative molecular biophysical morphometry. Deep learning single-cell nuclear segmentation (StarDist 2D) was used to compare cell density and nuclear area across the three implantation routes. Data represent mean ± SEM. *n* = 3 mice/group, (averaging 3 fields of view/mouse). Route comparisons were evaluated separately for cell density and mean nuclear area using one-way ANOVA with Tukey’s post hoc multiple comparisons test; observed trends in cellular density and nuclear compression did not reach statistical significance (*p* > 0.05).

OVCAR3 xenografts were established via intraperitoneal (IP), intrabursal (IB) and subcutaneous (SC) routes and monitored over route-specific time courses (Figure 1A). Tumour establishment, histology and marker expression were then compared across the three models.

IP and IB implantation achieved 100% engraftment across the sample cohorts. SC implantation achieved 83% engraftment (15/18 mice), with non-engraftment apparent by Week 4 on palpation (*n* = 1 in the Week 4 endpoint cohort, *n* = 1 in Week 6, and *n* = 1 in Week 9).

Having confirmed consistent engraftment in orthotopic routes, we next assessed whether HGSOC lineage identity was maintained across implantation sites. Histopathological and immunofluorescence assessment showed PAX8, WT1 and Ki67 expression across representative tumours from all three models (Figure 1B–D). The IB and IP models most closely recapitulated the distinct architectural hallmarks of HGSOC. In the IP model, H&E staining revealed a dense, globular morphology where PAX8 and WT1 expression was concentrated at the infiltrative tumour front, effectively mirroring late-stage disease (Figure 1, top row). The IB model best captured the primary architectural complexity of HGSOC, displaying organised glandular structures with uniform lineage marker expression throughout the ovarian stroma (Figure 1, middle row).

In contrast, the SC model represents a departure from the infiltrative nature of HGSOC and was defined by a dense, host-derived stromal surround. WT1 signal was reduced in SC sections, consistent with lower cellular density within fields dominated by the non-malignant stromal capsule (Figure 1, bottom row; Figure 1F). Histological examination revealed sharply circumscribed epithelial lobules with prominent internal fibrous banding and sporadic infiltration of large macrophage-like cells with abundant cytoplasm and rounded nuclei, consistent with host stromal encapsulation in the subcutaneous niche. Robust Ki67 positivity across all sites confirmed high proliferative capacity regardless of implantation niche (Figure 1E).

Notably, quantitative morphometric analysis revealed considerable architectural shifts across the three niches (Figure 1F). The IB tumours exhibited the highest cellularity, with a mean density of 1087 cells/mm^2^, an approximate two-fold increase over the SC model (583 cells/mm^2^) and the IP model (566 cells/mm^2^). This increased density was accompanied by a marked reduction in nuclear size. IB tumour nuclei were smaller (mean area 22 µm^2^) compared to those in the SC (43 µm^2^) and IP (57 µm^2^) environments.

The mean differences in cell density and nuclear area did not reach statistical significance (*p* > 0.05).

### 2.2. Implantation Site Governs Disease Kinetics and Spatiotemporal Dissemination

**Figure 2:**
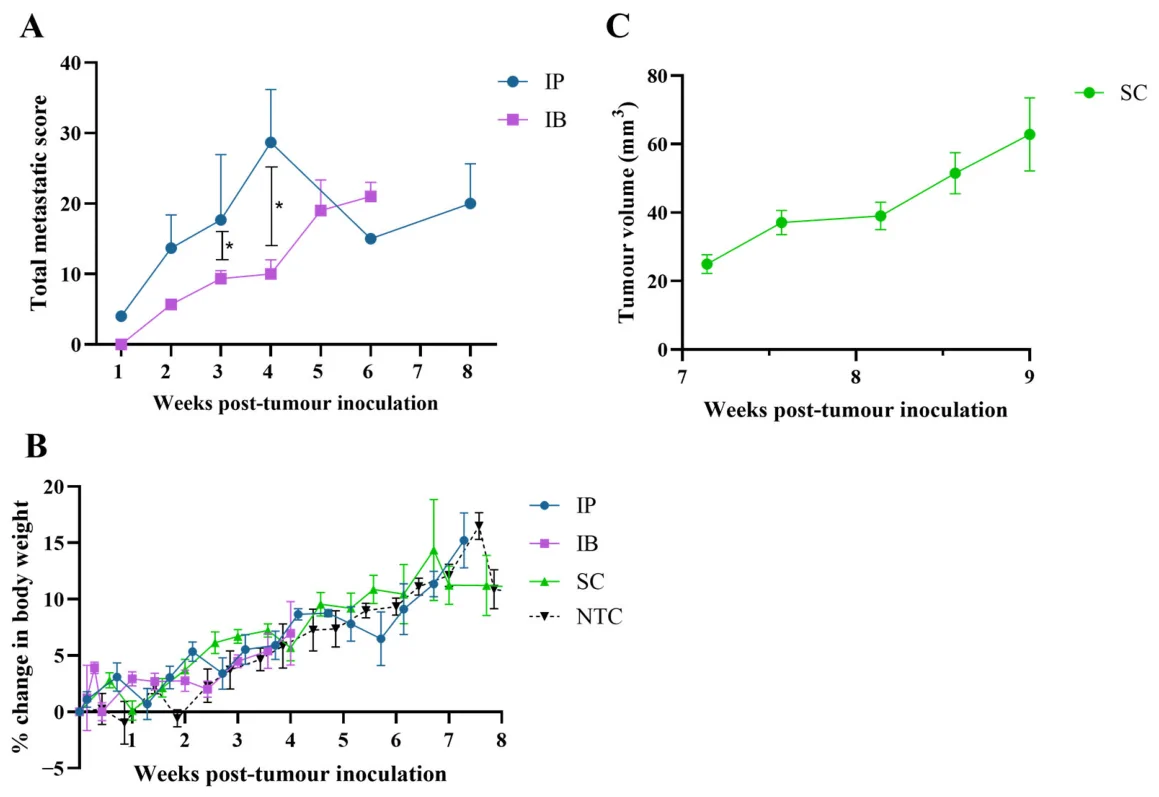
Site-specific disease kinetics and systemic physiological impact of OVCAR3 xenografts. (**A**) Cumulative peritoneal disease kinetics. Quantification of total metastatic burden demonstrating accelerated, exponential dissemination in the intraperitoneal model (IP, blue) versus delayed, compartmentalised kinetics in the intrabursal model (IB, purple), reflecting microenvironmental constraints on local growth versus peritoneal spreading. (**B**) Relative change in body weight. Data are expressed as a percentage of the initial weight (Day 0 = 100%). All cohorts maintained stable body mass throughout the study comparable to non-tumour controls (NTC, age-matched naïve mice, black dashed line). (**C**) Subcutaneous (SC, green) tumour expansion. Volumetric growth of SC OVCAR3 xenografts was monitored via calliper measurements (mm^3^). Data shows a steady, linear growth rate characteristic of the subcutaneous route. All data represent mean ± SEM (*n* = 3 mice/group/time point). Statistical significance was determined by two-way ANOVA with Tukey’s post hoc test (* *p* < 0.05).

The IP model exhibited the most aggressive progression profile, with an exponential increase in total metastatic score reaching peak levels by Week 4 (Figure 2A). After Week 4, the mean score reached a plateau. Histological sections from later time points showed regions of central acellularity, suggesting that central tumour necrosis in large masses may contribute to this kinetic shift alongside a transition toward diffuse, microscopic seeding, leading to an underestimation of the total viable tumour load based on the macroscopic scoring rubric alone [7]. Total ascites volume remained low, with an average of 400 µL first detected in the Week 8 cohort. Despite the high tumour burden, the IP model did not induce systemic cachexia, as mean body weights remained stable or increased throughout the 8-week timeframe relative to age-matched non-tumour-bearing naïve controls (Figure 2B).

The IB model produced a more controlled trajectory of local invasion. All mice developed macroscopic tumours by Week 3. While OVCAR3 cells eventually colonised the peritoneal cavity, the total metastatic score remained significantly lower than the IP model for the initial four weeks of the study (Figure 2A, *p* < 0.05 week 3 and week 4). Similar to the IP cohort, IB mice maintained stable body weights with no evidence of systemic morbidity throughout the 6-week period (Figure 2B). The sequestration of disease within the right ovary during the first four weeks suggests that the ovarian bursa imposes a significant barrier before widespread dissemination occurs.

In SC-implanted mice, tumours were palpable by Week 4 and increased steadily from Week 7 onward, reaching maximum tumour volumes of 173 mm^3^ by Week 9 (Figure 2C). The SC model was unique in its low inter-animal variability and lack of clinical distress or weight loss (Figure 2B). At necropsy, SC tumours were firm and well-demarcated from the underlying musculature.

**Figure 3:**
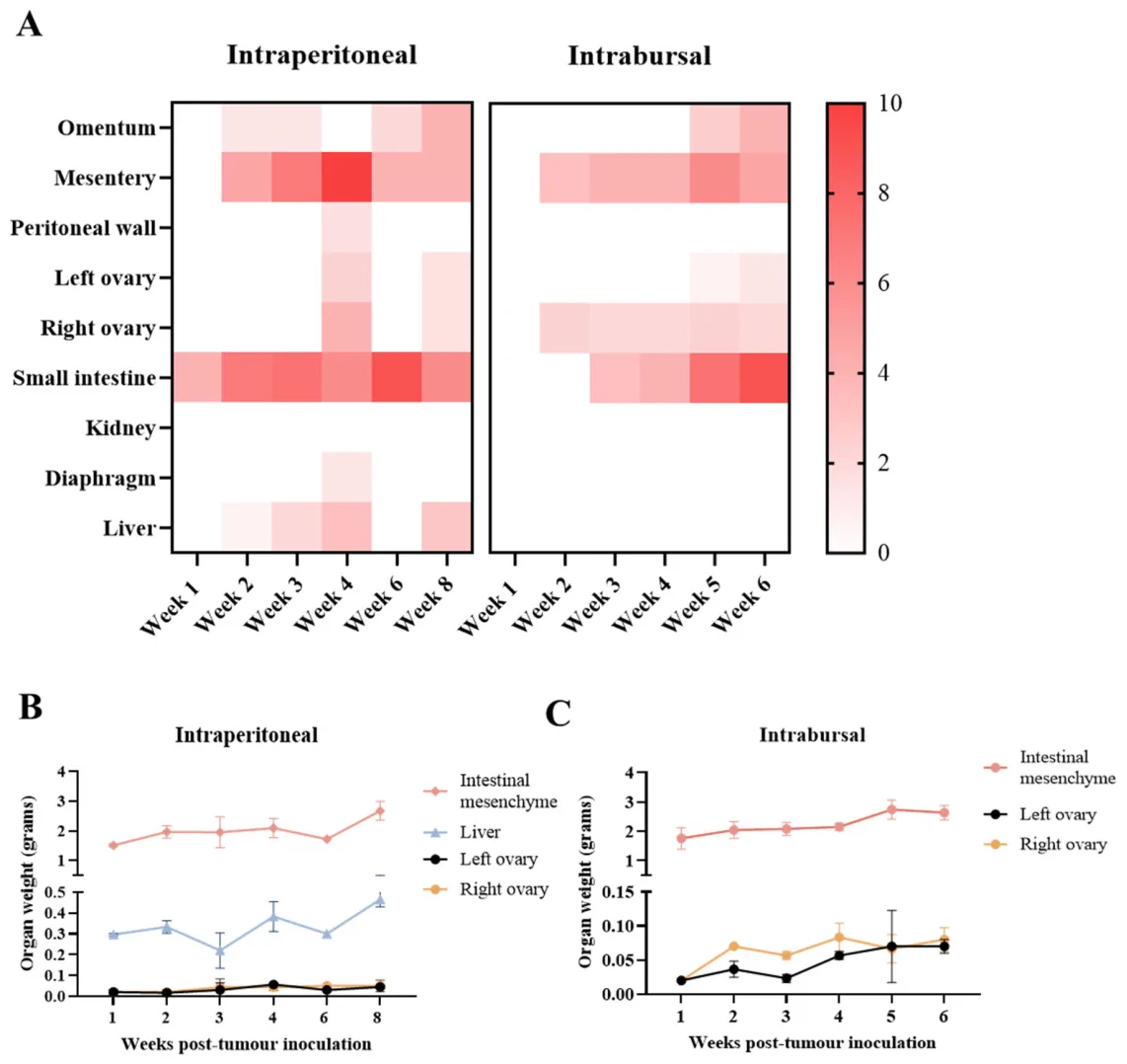
Anatomical tropism, organ-specific kinetics and spatiotemporal mapping of OVCAR3 dissemination. (**A**) Spatiotemporal heatmap of tumour scores. Semi-quantitative scores for nine anatomical sites are shown for the IP and IB models across all analysed weeks. The colour scale represents the mean score, from white (no disease) to deep red (maximum burden). The IP model shows rapid, global peritoneal colonisation, while the IB model maintains a localised signature focused on the reproductive tract before spreading to the intestinal mesentery. (**B**) IP model organ weight kinetics. Longitudinal weights (g) for the intestinal mesenchyme, liver, and bilateral ovaries. (**C**) IB model organ weight kinetics. Longitudinal weights (g) for the intestinal mesenchyme and bilateral ovaries. These distinct organ weight trajectories and spatiotemporal heatmaps reflect how local tissue architecture dictates initial tumour retention versus rapid abdominal tropism. All line graph data represent mean ± SEM.

Spatiotemporal analysis of tumour burden across nine peritoneal sites revealed that the primary implantation niche governs metastatic patterning. The IP model successfully recapitulated advanced peritoneal disease, with macroscopic nodules visible on the intestinal serosa and omental fat by Week 2. By Week 4, diffuse seeding across the mesenteric folds progressed to extensive omental involvement by Week 8 (Figure 3A). Quantitative organ weight analysis confirmed a marked increase in the mass of the intestinal mesentery and liver capsule (Figure 3B).

The IB model demonstrated a localised growth pattern with a defined window of delayed peritoneal spread. Tumours within the primary right ovary appeared as grey-white, lobulated masses. Metastatic patterning was primarily directed toward the contralateral left ovary, where score increases remained negligible until Week 5, at which point measurements became comparable to the primary site, reflecting late-stage contralateral metastasis (Figure 3C). Beyond the reproductive tract, regional mesenteric involvement was detectable but remained modest compared to the IP model (Figure 3A). Upper abdominal sites including the diaphragm and kidney remained largely quiescent, and no ascites was observed throughout the 6-week IB study.

The SC model remained entirely localised with no evidence of visceral involvement. Histopathology confirmed the presence of epithelial lobules confined within a stromal capsule and necropsy revealed a complete absence of metastatic spread or ascites.

## 3. Discussion

The primary finding of this study is that OVCAR3 engraftment, disease kinetics and tumour architecture differed among the three implantation routes. IP and IB implantation achieved 100% engraftment in the sampled cohorts whereas SC implantation achieved 83% engraftment. IP tumours disseminated rapidly throughout the peritoneal cavity, IB tumours initially developed near the ovarian site before later dissemination, and SC tumours remained localised. These differences have practical implications for model selection and experimental planning.

Immunofluorescence staining confirmed the consistent expression of the HGSOC lineage markers PAX8 and WT1 across all models. While spatial immunofluorescence revealed site-dependent marker topology by concentrating specifically at the invasive tumour front in IP dissemination versus displaying uniform, glandular organisation in orthotopic IB masses, the overall conservation of this Müllerian molecular profile reinforces that the observed morphological and TME differences are driven by the implantation site rather than genetic drift or clonal selection [6,8,9]. Historically, cell lines like SKOV3 and A2780 have dominated ovarian cancer research; however, modern genomic re-evaluations have highlighted that SKOV3 lacks the *TP53* mutations and extensive copy number variations (CNVs) that are near-ubiquitous hallmarks of HGSOC [9,10,11].

OVCAR3 has published genomic features associated with HGSOC [12], although *TP53* and copy number status were not independently assessed in the experimental culture used in this study.

SC tumours remained localised and were surrounded by dense stromal tissue, with occasional large cells displaying macrophage-like morphology. As immune cell markers were not assessed, the identity and function of these cells could not be determined [13]. No visceral dissemination or ascites was observed in the SC model. The external tumour location allowed non-invasive monitoring by calliper and straightforward collection at necropsy. Engraftment occurred in 15 of 18 mice, and this rate should be considered when determining initial cohort size. Although the SC model provides a practical approach for monitoring local tumour growth, drug penetration, pharmacokinetics, treatment response and cellular therapy trafficking were not evaluated in this study [14].

The IB model produced glandular tumour architecture and an initial period of growth near the ovarian site before contralateral and peritoneal spread. Quantitative deep learning single-cell morphometry (StarDist 2D) demonstrated considerable nuclear compression and higher cellular density in the IB niche relative to the peritoneal cavity, highlighting the degree of solid stress and mechanical confinement imposed by the bursal membrane [15]. The key functional feature of this model is the four-week window of localised ovarian disease before contralateral spread, as demonstrated by the spatiotemporal scoring data (Figure 3C). This defined latency period makes the IB model uniquely suited for studying the early steps of invasion and bursal breach that precede peritoneal dissemination, events that are not observable in the IP model, where disease is already disseminated at the time of first assessment. The 100% engraftment observed in the IB cohorts demonstrates the feasibility of the procedure in this experimental series, provided microsurgical technique limits leakage into the peritoneum at injection [16].

The IP model exhibited rapid dissemination to the omentum, and other peritoneal sites consistent with the seed-and-soil pattern of ovarian cancer dissemination [17].

Increases in intestinal mesentery and liver capsule mass were observed from Week 4 (Figure 3B), and ascites was detected at the later endpoint. The first two weeks captured early peritoneal dissemination under this protocol, although mesothelial adhesion and other mechanisms of metastatic colonisation were not measured directly.

Published studies vary in their reporting of OVCAR3 engraftment [9,18]. In the present study, the three routes used different inocula and procedures: SC, IB and IP mice received 5 × 10^5^, 1 × 10^6^ and 5 × 10^6^ cells, respectively. Consequently, the lower SC engraftment rate cannot be attributed to implantation site alone, and the cause of individual SC engraftment failures cannot be determined from these data. Reporting both enrolled and engrafted animal numbers provides useful information for cohort sizing and comparison with future studies.

The kinetic data identified different observation periods for the three protocols. IP tumours developed disseminated peritoneal disease within two weeks of inoculation, whereas IB tumours remained predominantly localised near the ovarian site for four weeks before later spread. SC tumours allowed for longitudinal monitoring of local growth. These timelines may assist endpoint selection, although the most appropriate intervention period will depend on the specific research question.

Several limitations should be considered. OVCAR3 is a long-established cell line derived from malignant ascites, and a single cell line cannot represent the inter-patient and intratumour heterogeneity of HGSOC. The models did not incorporate 3D culture-derived spheroids or the complete cellular and extracellular matrix components of the human tumour microenvironment. Athymic nude mice lack mature T-cell immunity, and immune cell populations were not characterised in this study. PAX8, WT1 and Ki67 constitute a limited marker panel, and transcriptomic, broader protein, immune recognition and therapeutic target profiling were not performed. Evaluation in additional cell lines, patient-derived models and independent experimental cohorts would extend the present findings.

**Figure 4:**
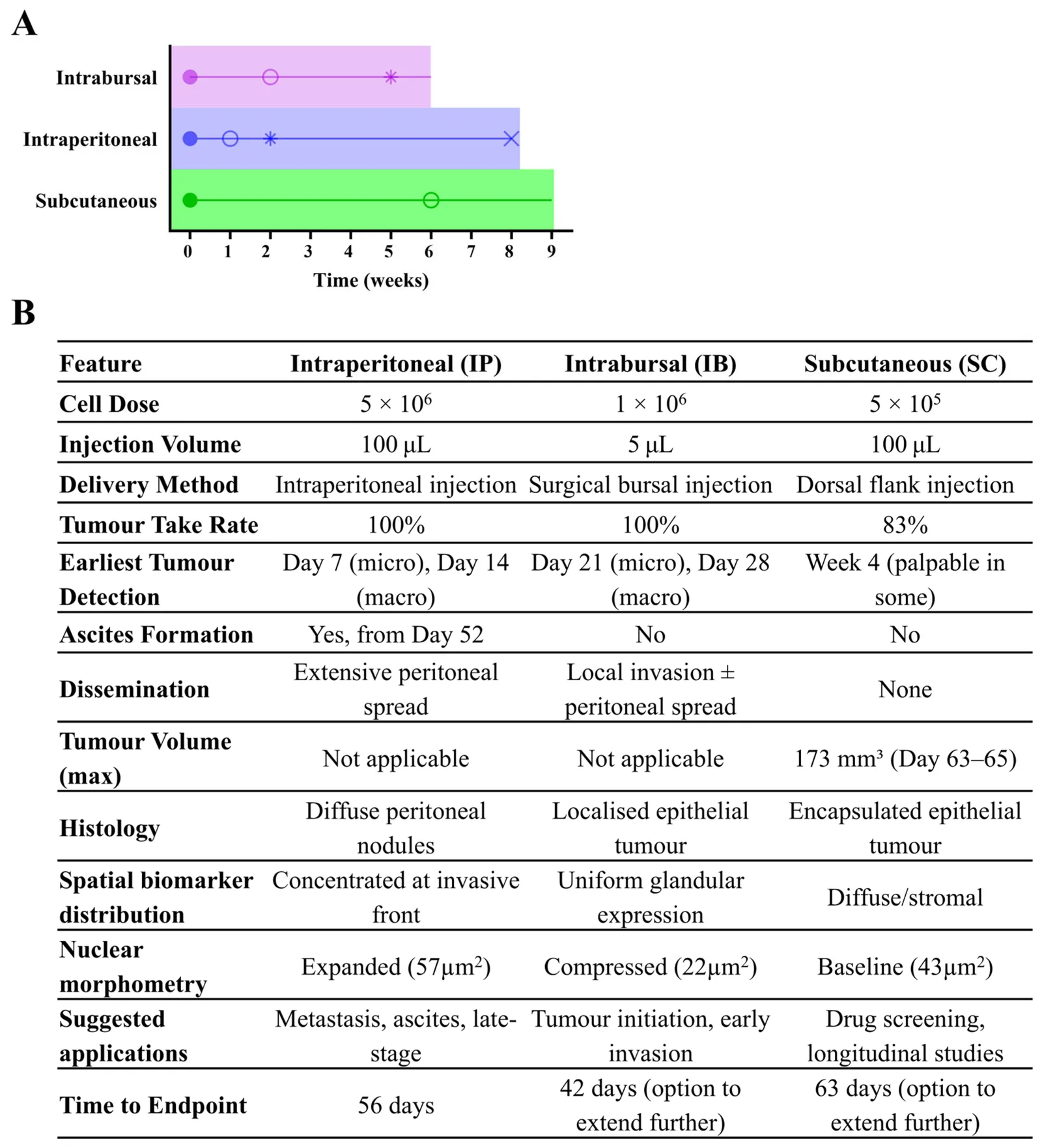
Spatiotemporal milestone timeline and comparative summary matrix for OVCAR3 xenograft routes. (**A**) Spatiotemporal progression timeline. A comparative kinetic map illustrating the distinct windows of disease latency and spread across the three OVCAR3 models over a 9-week period. Key clinical milestones are denoted to facilitate experimental planning: ● = Cell inoculation; ○ = Initial presentation of disease (first non-zero tumour score); ∗ = Evidence of metastatic dissemination; × = Onset of malignant ascites. This timeline identifies the optimal intervention windows for early-stage (IB) versus advanced-stage (IP) investigations. (**B**) Summary matrix integrating empirical measurements (cell dose, engraftment rate, dissemination pattern, and nuclear morphometry) across SC, IB, and IP routes to optimise model selection and assist in experimental planning.

Model selection requires balancing biological relevance against practical considerations. SC implantation enables non-invasive monitoring of local tumour growth, IB implantation provides a period of ovarian region tumour development before dissemination, and IP implantation produces rapid disseminated peritoneal disease. Figure 4 summarises the measured engraftment frequencies, progression timelines and practical characteristics of each route. These observations can guide model and endpoint selection, while specific pharmacological and mechanistic applications require direct validation in the intended experimental setting.

## 4. Materials and Methods

### 4.1. Cell Culture

The human ovarian cancer cell line NIH:OVCAR-3 (ATCC HTB-161) was maintained in RPMI-1640 medium supplemented with 20% foetal bovine serum (Bovogen Biologicals, Melbourne, VIC, Australia), 100 U/mL penicillin, 100 μg/mL streptomycin, and 0.01 mg/mL bovine insulin (Sigma-Aldrich, St Louis, MO, USA). Cells were maintained at 37 °C in a humidified 5% CO_2_ incubator and passaged at 70–80% confluence. For all in vivo experiments, cells were trypsinised and resuspended in a 1:1 mixture of PBS and growth factor-reduced Matrigel (Corning Inc., Corning, NY, USA) on ice. Cell viability exceeded 90% as determined by trypan blue exclusion. All murine injections were performed immediately after preparation. Cell-line identity was confirmed by STR genotyping performed by the Australian Genome Research Facility (AGRF, Melbourne, VIC, Australia; order ABMS-15478). *TP53* status was reported from public genomic database characterisations of OVCAR3 [9,11] and was not independently determined in the experimental culture

### 4.2. Animals

Nulliparous BALB/c nude female mice (BALB/c-Foxn1^nu^; Ozgene, Perth, WA, Australia), aged between 6 and 8 weeks and weighing 18–22 g were acclimatised for 7 days prior to procedures. Mice were ear-notched for identification and housed in groups of 3–5 per cage with ad libitum access to food and water. All animal procedures were conducted in accordance with the Australian Code for the Care and Use of Animals for Scientific Purposes. Mice were housed in specific-pathogen-free (SPF) conditions at the Monash Health Translational Precinct (MHTP) Animal Facility and monitored daily for health and welfare. Humane endpoints were strictly adhered to and monitored in accordance with institutional guidelines.

Mice were randomly allocated into three experimental groups representing intraperitoneal (IP), intrabursal (IB), and subcutaneous (SC) tumour models. Each group consisted of 15–18 mice, subdivided into 5–6 timepoint cohorts (*n* = 3 per endpoint). Non-engrafted animals (defined by the absence of palpable or macroscopic tumour mass by Week 4) were excluded from downstream analyses. Fixed-interval endpoints (Weeks 1–9) were established based on preliminary kinetic pilots to capture the full spectrum of HGSOC progression, ranging from early local confinement to advanced ascites formation and end-stage carcinomatosis. Non-tumour-bearing age-matched naïve mice served as baseline body weight controls (*n* = 3 per endpoint).

### 4.3. Intraperitoneal (IP) Tumour Model

Mice (*n* = 18) received an intraperitoneal injection of 5.0 × 10^6^ OVCAR3 cells suspended in 100 μL of 50% Matrigel/PBS using a 29 G insulin syringe. Injections were performed in the lower right abdominal quadrant with the animal gently restrained using the scruffing technique. Mice were monitored for 20 min post-injection before returning to housing. Abdominal circumference was recorded biweekly upon the formation of ascites. Animals were euthanised at Weeks 1, 2, 3, 4, 6 and 8.

### 4.4. Intrabursal (IB) Tumour Model

Mice (*n* = 18) underwent surgical implantation of 1.0 × 10^6^ OVCAR3 cells in 5 μL of 50% Matrigel/PBS into the right ovarian bursa as described previously [19]. Briefly, 0.1 mg/kg buprenorphine and 5 mg/kg meloxicam were injected subcutaneously prior to surgery as analgesics. Mice were anaesthetised with 5% isofluorane in 1 L/min oxygen in an induction chamber then maintained at 2–2.5% isofluorane in 0.3 L/min oxygen using a precision nosecone. The skin was excised at the mid-dorsal region, and the peritoneal membrane was latero-dorsally excised at the position of the right ovary. The ovarian fat pad was externalised and stabilised using a micro-serrefine clamp. OVCAR3 cells were injected directly into the bursal sac using a 30 G Hamilton syringe. The incision was closed using two Michele suture clips, which were removed 7–10 days following surgery. Mice received postoperative carprofen (18 mg/mL in drinking water) and were monitored twice daily for 48 h, then twice weekly. Animals were euthanised at Weeks 1, 2, 3, 4, 5, and 6.

### 4.5. Subcutaneous (SC) Tumour Model

Mice (*n* = 18) received a subcutaneous injection of 5.0 × 10^5^ OVCAR3 cells in 100 μL of 50% Matrigel/PBS into the left dorsal flank using a 28 G needle. Tumour formation was assessed by palpation and longitudinal growth was quantified via live calliper measurements twice weekly. Tumour volume was calculated using the formula (length × width^2^)/2. Animals were euthanised at Weeks 1, 2, 3, 4, 6 and 9 and tumours were excised at necropsy.

### 4.6. Tissue Collection and Histology

Mice were sacrificed at predefined endpoints for euthanasia via CO_2_ inhalation. Ascitic fluid, when present, was withdrawn using a 21 G syringe and measured. Major organs (liver, spleen, kidneys, lungs, intestines, ovaries, brain) and tumour tissues were harvested, weighed, and fixed in 10% neutral-buffered formalin for at least 24 h. Tissues were processed, embedded and Haematoxylin and Eosin (H&E) stained by the Monash Health Translation Precinct (MHTP) Histology Platform, Clayton, Australia.

Independent veterinary pathology review was conducted on a subset of formalin-fixed, paraffin-embedded tissues spanning all three models and timepoints. H&E-stained sections were evaluated by a board-certified veterinary pathologist for evidence of tumour localisation, organ invasion, inflammation, necrosis, and incidental background pathology. Observations from this review informed representative image selection and confirmed the absence of systemic or metastatic disease outside the peritoneal cavity.

### 4.7. Tumour Scoring (IP and IB Models)

Tumour burden at necropsy for internal dissemination models (IP and IB) was assessed across multiple organs using a semi-quantitative scoring system (Table 1) adapted from established ovarian cancer histopathology protocols [7]. For each tissue site (e.g., ovary, omentum, diaphragm, mesentery, liver), (a) the tumour volume was designated a score of either 0, 1, 2, or 3 depending on approximate nodule size in mm, and (b) the tumour dissemination score was assigned according to a scale of 0–5 depending on the number of visible nodules manually counted. To ensure scoring reliability, all tissue evaluations were performed with investigator blinding to experimental groups, and a subset of samples (20%) underwent independent repeat scoring to confirm high inter-rater concordance.

### 4.8. Immunofluorescence

Immunofluorescence was performed as described [20] on 4 μm formalin-fixed paraffin embedded (FFPE) tumour tissues. Following deparaffinisation of FFPE tissues in histosol and graded alcohol, antigen retrieval was performed by HIER (10 mmol/L Tris, 1 mmol/L EDTA, pH 9.0). Non-specific binding was blocked by 5% BSA/PBS with 10% goat serum and primary antibodies were incubated at 4 °C overnight in a humidified chamber. Primary antibodies used were Ki67 (1:300, Cell Signaling Technology, Danvers, MA, USA; #9129), PAX8 (1:100, Proteintech, Rosemont, IL, USA; #10336-1-AP) and WT1 (1:100, Abcam, Waltham, MA, USA; #ab89901). Secondary antibodies (1:200) were incubated for 2 h at room temperature in a humidified chamber: Goat anti-Rabbit IgG (H + L) Alexa Fluor 488 (Thermo Fisher Scientific, Waltham, MA, USA) and Goat anti-Rabbit IgG (H + L) Alexa Fluor 647 (Thermo Fisher Scientific). Positive control tissues were not included; target-expressing OVCAR3 tumour tissue served as the internal positive control for PAX8, WT1, and Ki67, with signal specificity confirmed by parallel secondary-only and matched concentration Rabbit IgG isotype controls (Supplementary Figure S1). Following secondary antibody incubation, nuclei were counterstained with DAPI (D21490, Thermo Fisher Scientific), samples were washed in PBS and then counterstained with Sudan Black solution (Sigma Aldrich, Bayswater VIC, Australia) (0.3% in 70% ethanol) to reduce intrinsic autofluorescence. Slides were mounted using ProLong™ Gold Antifade Mountant (P36930, Thermo Fisher Scientific). Fluorescence images were captured on the Cytation 3 Multimode Imager (BioTek Instruments, Winooski, VT, USA), and digital image analysis was performed using Gen5 software (v3.08; BioTek Instruments).

### 4.9. Data Analysis

Tumour volumes, ascites volumes, organ weights and body weights were recorded in Microsoft Excel and visualised graphically using GraphPad Prism v10.6.1 (GraphPad Software Inc., San Diego, CA, USA). Clinical signs and tumour scores were logged according to approved animal monitoring sheets. Total metastatic scores were calculated as follows: total metastatic score = dissemination score × tumour volume score. For tumour scores and organ weights, means were compared using either an unpaired *t*-test or one-way ANOVA as indicated. Nuclear segmentation and density quantification (cells/mm^2^) were performed on H&E-stained sections using the StarDist 2D deep learning model within QuPath (v.0.7.0) [21,22]. Morphometric analysis was conducted in QuPath by calculating the mean nuclear area (µm^2^) for all detected objects within the annotated tumour regions to assess cellular compression and mechanical constraint. Comparisons across implantation routes were evaluated separately for cell density and mean nuclear area using one-way ANOVA with Tukey’s post hoc test.

## 5. Conclusions

No single OVCAR3 model recapitulates the full spectrum of HGSOC. Our findings demonstrate that anatomical niches are associated with distinct structural, kinetic and spatial marker profiles vivo. In summary, IP implantation produces rapid peritoneal dissemination and ascites; IB implantation captures localised growth prior to peritoneal spread; and SC implantation provides localised, calliper-measurable growth. The engraftment rates, progression timelines, and spatiotemporal profiles reported here provide an empirical reference for endpoint timing and cohort planning. The persistent inconsistency in how OVCAR3 take rates are reported across the literature has created uncertainty about model reliability; the transparent reporting of both successful and failed engraftments in this study directly addresses that gap.

## Figures and Tables

**Table 1:** Tumour scoring parameters.

Tumour Volume Score						
Nodule diameter (mm)	0	<0.1	0.1–<0.5	>0.5		
Score	0	1	2	3		
Dissemination Score						
Number of nodules	0	1	2–5	5–10	10–20	>20
Score	0	1	2	3	4	5

## Data Availability

The raw data supporting the conclusions of this article will be made available by the authors on request.

## Supplementary Materials

The following supporting information can be downloaded at: https://www.mdpi.com/article/10.3390/ijms27187981/s1.

## Abbreviations

The following abbreviations are used in this manuscript:

HGSOC	High-grade serous ovarian cancer
TME	Tumour microenvironment
PDX	Patient-derived xenograft
CDX	Cell line-derived xenograft
SC	Subcutaneous
IP	Intraperitoneal
IB	Intrabursal
CNV	Copy number variation
TCGA	The Cancer Genome Atlas
