# Supplementary material for: Comparative Characterisation of Subcutaneous, Intraperitoneal and Intrabursal OVCAR3 Models
Source: Int J Mol Sci. 2026 Sep 8;27(18):7981. doi: 10.3390/ijms27187981 (PMC13606854; doi:10.3390/ijms27187981)
Supplement: Supplementary file 1 [file ijms-27-07981-s001.zip › ijms-4481877-supplementary.pdf]

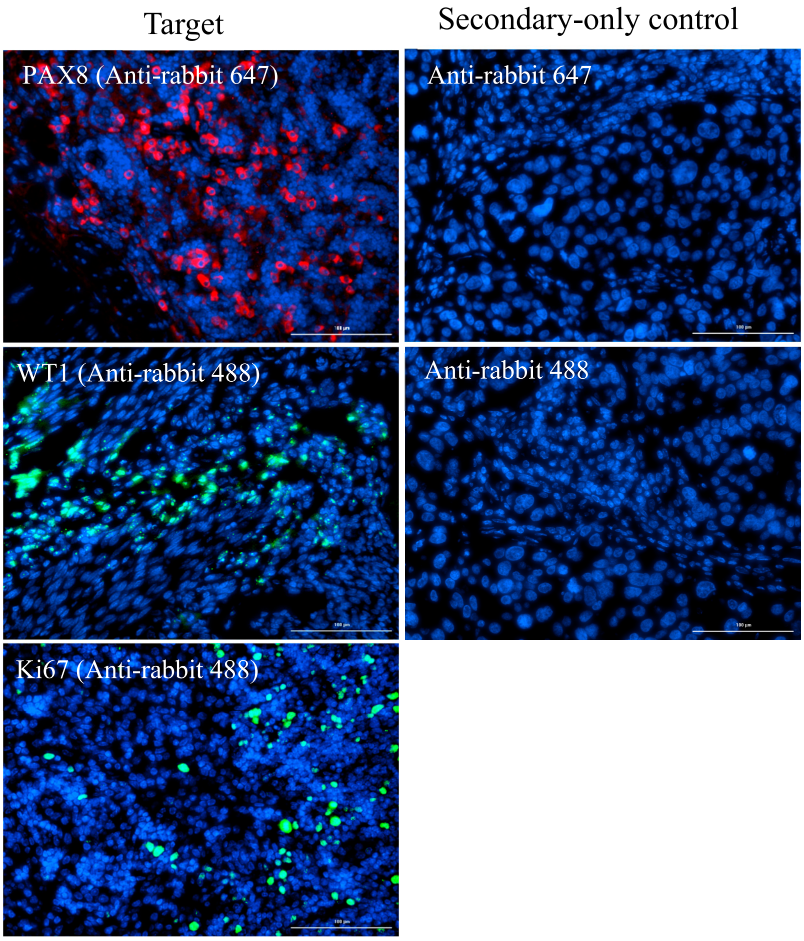

Supplementary Figure S1. Secondary-only controls for immunofluorescence. Representative immunofluorescence images of OVCAR3 xenograft tumour tissue sections showing target marker staining alongside parallel secondary-only controls. Primary target antibodies (left panels: PAX8, WT1, Ki67) were detected using fluorophore-conjugated secondary antibodies as indicated. Control sections (right panels) were processed under identical incubation, washing, and imaging parameters with primary antibodies omitted. Nuclei were counterstained with DAPI (blue). Scale bars = 100 μm. Note: The Anti-rabbit 488 secondary-only control panel serves as the negative control for both WT1 and Ki67 target channels.
